# Conservative management of low-risk papillary thyroid carcinoma: a review of the active surveillance experience

**DOI:** 10.1186/s13044-023-00148-6

**Published:** 2023-03-13

**Authors:** Anabella Smulever, Fabian Pitoia

**Affiliations:** Division of Endocrinology, Hospital de Clínicas, University of Buenos Aires, Córdoba 2351, 5th floor, Buenos Aires, Argentina

**Keywords:** Active surveillance, Low risk, Papillary thyroid carcinoma, Observation, Papillary thyroid microcarcinoma, Thyroid cancer, Indeterminate thyroid nodules

## Abstract

The detection of low-risk thyroid carcinoma has increased in recent decades, although disease-specific mortality remained without changes. The high prevalence of occult carcinomas in autopsy studies, and hence the underlying indolent course of this entity, prompted the emergence of active surveillance as an alternative approach to these tumors. This strategy aims to recognize the minority group of patients who will develop clinical progression and probably benefit from deferred surgery. Experience around the world has shown that during active surveillance these tumors are mostly unchanged in size, with very-slow growth and even a decrease in diameter. Moreover, the rates of lymph node metastases were low and easily handled by rescue surgery, and distant metastases have not been reported. Given the high prevalence of small thyroid carcinomas and the excellent outcomes for observation, active surveillance provides a safe and feasible alternative in properly selected patients with low-risk thyroid cancer.

## Background

### Small papillary thyroid carcinoma: epidemiology and basis of active surveillance

Papillary thyroid carcinoma (PTC) is the most common well-differentiated thyroid cancer, with microcarcinoma as the most frequent form of presentation [1, 2]. According to the database provided by the National Cancer Institute from the United States of America, the incidence of these tumors has tripled over the last decades, probably as a result of overdiagnosis, with a low and stable mortality rate. Indeed, more than 60% of its incidence has been attributed to tumors smaller than 1 cm and it has been estimated that 1.2% of the population will be diagnosed with thyroid cancer in their lifetime [3, 4]. The natural history of these tumors often exhibits stability or slow-growing, or may even shrink [3]. This was illustrated by several autopsy studies worldwide showing a high rate of occult thyroid carcinomas, with a prevalence of up to 35.6% [5–7], representing 100 to 1000 times more than clinical carcinomas [8]. From another point of view, patients with papillary thyroid microcarcinomas (PMCs) who undergo total thyroidectomy, with or without radioiodine ablation, have a risk of recurrence at 10–12 years ranging from 0.5% to 1% for a single focus and increases to 5% when multiple foci or clinical lymph node metastases are initially diagnosed [9, 10]. Distant metastases are estimated to occur with a frequency of less than 1% [11], although there are currently no real-life studies that demonstrate their existence in patients under active surveillance, it has been shown that the prevalence in low-risk thyroid cancer would not be larger than 0.5% [12, 13]. Therefore, most PMCs usually are incidentally found, with indolent development, and it does not lead to health and life hazards [8–10, 14]. Under these premises, over the last decades, active surveillance has been taking a leading role in the management of these tumors. This strategy is based on the correct selection and follow-up of patients with small low-risk papillary carcinoma, providing, if necessary, the appropriate surgical intervention at the right moment, optimizing therapeutic resources, and minimizing adverse events [15, 16].

This review aims to summarize the strategy of active surveillance according to current international evidence, to characterize the factors which impact the decision of this approach and the proper selection of patient candidates, and to discuss new insights into active surveillance.

### Outcomes of patients under active surveillance: trials around the world

In 1993, the first clinical trial on active surveillance in papillary thyroid microcarcinoma was conducted in Kuma Hospital in Japan by Professor Miyauchi [17]. While preliminary results were shown briefly in 2003 [17], in 2014, Ito et al. reported that from 1235 patients enrolled with PMC in 10 years of follow-up, 8% exhibited an increase in 3 mm in a larger diameter, 3.8% metastatic lymph nodes and none had distant metastases or disease-related death [18]. Almost simultaneously, Dr. Sugitani from the Cancer Institute Hospital in Tokyo, conducted an AS prospective study, gathering similar results, even when later included T1b tumors in this practice [19, 20]. The first prospective AS study in America was published in 2017, by Tuttle et al. [21] from the Memorial Sloan Kettering Cancer Center Hospital in the United States. From 284 patients with tumors up to 1.5 cm in maximum diameter under AS, an increase in diameter ≥ 3 mm was detected in 3.8% of patients, with a cumulative incidence of 2.5% at 2 years and 12.1% at 5 years. In contrast, 12.7% showed an increase in tumor volume ≥ 50% [21]. In Latin America, some studies show that this approach can be carried out in centers with high experience in the management of patients with thyroid cancer [22–24]. The first Argentine report was carried out from 2014 to 2018 and included 137 patients who attended the Hospital de Clínicas [22]. From 34 eligible patients who accepted AS, the frequency of tumor enlargement was 17% after a median of 4.6 years of follow-up, without any evidence of nodal or distant metastases [22]. In 2020, we updated data from our cohort evidenced a reduction in tumor growth rate to 14.4%, and we were the first in Latin America to report a 4.8% occurrence of lymph node metastases after a median follow-up of up to 4 years. Twelve of forty-one patients underwent deferred surgery after a mean of 2.9 years of AS, and none of them had evidence of disease after 3.5 years of the surgery [25]. In Colombia, Sanabria et al. recently reported a 10.8% of tumor growth, with a mean of 12 months follow-up [23].

Finally, a number of meta-analyses with mixed results are available. Cho et al*.* [26] estimated that the median increase in tumor diameter > 3 mm during AS was 5.3% (range, 4.4–6.4) and the occurrence of lymph node metastases was 1.6% (range, 1.1–2.4)], which are similar to the results calculated by Saravana-Bawan et al. [27]. Aryanti et al. recently showed that the pooled proportion of cases of increased tumor size > 3 mm during active surveillance was 12% and of the development of lymph node metastases was 4.9% [28]. Regardless of these nuanced differences that probably relate to the design and included studies, none of the current clinical trials reported distant metastases or cancer-related deaths as a consequence of undergoing active surveillance in patients with low-risk papillary carcinomas. The main clinical trials are listed in Table 1.

**Table 1:** Main clinical trials of active surveillance in patients with low-risk papillary thyroid carcinomas

Cohort (year)	n	Median follow-up (months)	Tumor growth ≥ 3 mm (% patients)	Lymph node metastasis (% patients)	Distant metastases (% patients)	Deaths (% patients)
**Sugitani et al.** (2010) [19]	230	5	7	1	0	0
**Ito et al.** (2014) [18]	1235	120	8	3.8	0	0
**Oda et al.** (2016) [29]	1179	36	2.3	0.5	0	0
**Kwon et al.** (2017) [30]	192	30	14	0.5	0	0
**Tuttle et al.** (2017) [21]	291	25	3.8	0	0	0
**Kim et al.** (2018) [31]	126	26	5.6	0.8	0	0
**Sakai et al.** (2018) [20]	61	88.8	7	3	0	0
**Oh et al.** (2018) [32]	370	32.5	6.4	1.4	0	0
**Smulever & Pitoia** (2019) [22]	34	42	17	0	0	0
**Smulever & Pitoia** (2020) [25]	41	37	14.6	4.8	0	0
**Molinaro et al*.*** (2019) [33]	93	19	2.1	1	0	0
**Campopiano et al*.*** (2021) [34] (2021) [34]	109	31	4.5	2.7	0	0
**MaeSTro** (2022) [35]	755	41.4	5.4	1.3	0	0

### Implementation of AS

The concept of active surveillance has been increasingly attractive among clinicians over the last few years, understood as a close and dynamic tool that allows the detection of that minority group of patients with clinical progression who will benefit from rescue surgery [36]. However, certain controversies still exist concerning proper implementation, particularly the correct timing of diagnosis and follow-up, tumor progression criteria, and the correct indication for eventual rescue surgery. The recent consensus statements from the Japan Association of Endocrine Surgery Task Force (JAES) for the management of papillary thyroid microcarcinoma confirm that the starting point for active surveillance is to obtain a cytologic diagnosis to optimize patient adherence to AS program, providing reliability for research studies [37]. While a few exceptions may apply, the 2015 American Thyroid Association (ATA) guidelines recommend against performing fine-needle aspiration biopsy (FNAB) for sub-centimetric thyroid nodules, even with highly suspicious thyroid cancer ultrasonographic features, to minimize overdiagnosis and overtreatment [9, 21, 38].

Alternatively, we classify this action as “undercover active surveillance¨ [39]. This approach involves the chance of observing a thyroid nodule smaller than 1 cm with a suspicious ultrasound pattern (e.g., markedly hypoechogenic, with irregular borders and/or microcalcifications) without performing FNAB. After assessing if the patient is appropriate for active surveillance, it is essential to emphasize the high likelihood of malignancy (70–90%) [9]. If the decision is made not to perform FNAB, it will be followed as if under traditional active surveillance [2, 15]. The outcomes of this approach have been similar to traditional AS [22].

**Fig. 1:**
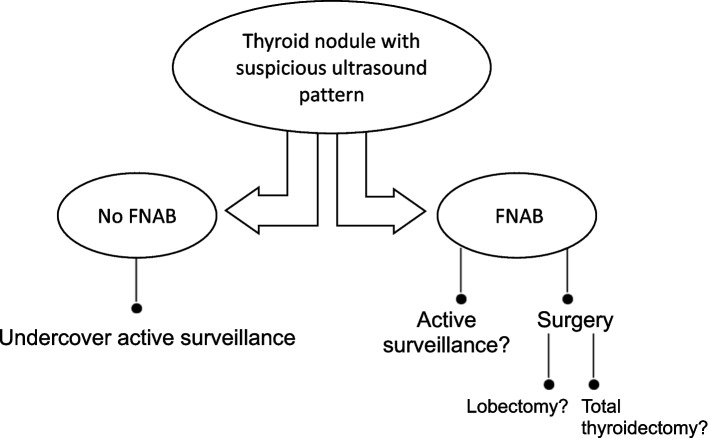
Proposed algorithm for the initial management of low-risk papillary thyroid carcinomas [39]. With ultrasound findings of a suspicious thyroid nodule, active surveillance (traditional or undercover) or immediate surgery may alternatively be offered as the first line of management, taking into account the variables related to the tumor, the patient, and the medical team

A proposed algorithm for the initial management of low-risk papillary thyroid carcinomas is shown in Fig. 1.

Although the correct interval between explorations is not uniform, most prospective studies have adopted it every six months with thyroid and neck ultrasound within the first two years and then once every one or two years if stability is documented [21, 37, 40]. These examinations aimed to assess disease progression, particularly changes in tumor size and the appearance of other thyroid lesions or lymph node metastases, probably throughout the patient´s lifetime [41]. In this regard, the definition of progression and the indication for conversion surgery is also a matter of discussion. Most prospective clinical trials consider the diagnosis of disease progression if the tumor grows 3 mm or more in diameter from baseline and there is evidence of extrathyroidal extension or lymph node metastases [18, 19, 37]. The JAES consensus states that if the tumor only exhibits an increase in size without other aggressive features, some patients may continue AS until it reaches 13 mm [37]. For its part, Tuttle et al. [21] reported that an increase in tumor volume of more than 50% precedes the enlargement of 3 mm or more in diameter, which may allow early diagnosis of progression, although this parameter might overestimate it, as some authors believe [42]. Thus, most long-term prospective studies did not report recurrent laryngeal nerve paralysis or distant metastases during AS using the diameter definition, showing that this is a simple and reproducible progression parameter [37].

### Factors on decision-making in AS

When deciding to perform active surveillance, one of the essential aspects is to properly select the candidate patient bearing a low-risk papillary thyroid cancer by assessing the risk factors for tumor progression [18, 43]. Thus, the latest JAES statements suggest that immediate surgery should be for patients who present: clinical lymph node or distant metastasis, invasion of the RLN or trachea or attached to these structures, diagnosis of an aggressive subtype of papillary thyroid carcinoma on cytology, and have another indication of neck surgery (other thyroid or parathyroid disease) [37]. This consensus also suggests that whether during active surveillance, there is a change in patient preference, the tumor diameter reaches 13 mm, new lymph node metastasis, or a new neck disease requiring surgery is found, rescue surgery is recommended [37].

Although scarce evidence is available from prospective studies, several predictive variables of tumor progression have been proposed, as listed below.

#### Tumor location

Even when tumors located in contact with the anterior thyroid pseudocapsule, including those with evidence of invasion of perithyroid muscles, may not necessarily be contraindications for AS. If rescue surgery is required, it would only involve a partial resection of the muscles with no further impact on the quality of life or on the oncologic prognosis. However, as mentioned above, tumors attached to the trachea or located along the path of the recurrent laryngeal nerve (RLN) are inappropriate for AS due to the potential tumor growth into these structures [18, 43, 44]. Ito et al*.* characterized an association between the angles formed by the tumor and tracheal surface with the chance of tracheal invasion [45]. In that study, 12 (24%) of 51 PMCs greater than 7 mm in maximal diameter were composed of obtuse angles and showed tracheal invasion that required resection of cartilage and mucosa, while none of the 286 PMCs that formed acute or almost right angles showed a significant tracheal invasion [45]. On the other hand, the risk of LRN invasion was associated with the absence of normal tissue interposed between the tumor and the thyroid surface in the direction of the nerve. Thus, 9% of 98 PMCs greater than 7 mm without normal tissue interposed required a partial or segmental LNR resection, while none of the 776 PMCs with normal borders exhibited microscopic LRN invasion. As expected, none of the microcarcinomas lower than 7 mm showed tracheal or LRN invasion [45].

#### Age at diagnosis

In contrast to what was demonstrated in more advanced stages of thyroid cancer in which older patients comprise a subgroup with a poorer prognosis, in the case of small low-risk tumors, the opposite is true. Prospective studies demonstrated that age is inversely proportional to the risk of disease progression, making older patients the best candidates for AS [16, 18]. Ito et al. informed the 10-year clinical progression rate in patients under 40 was 8.9%, while patients between 40 and 60 years of age and over 60 had progression rates of 3.5% and 1.6%, respectively [16]. Recently, this group estimated the lifetime probability of disease progression during AS for each age decade between the 20 s and 70 s [46]. Thus, in patients in their 20 s and 70 s, the probability of progression was 48.6% and 3.5%, respectively [46]. However, while younger patients are more likely to progress, only half will require rescue surgery during their lives, and probably none will show life-threatening recurrence or die from thyroid carcinoma [46]. Therefore, while older patients with low-risk thyroid carcinomas are ideal candidates for AS, younger patients may also be appropriate for this approach.

#### Serum thyrotropin levels

The role of ThyroidStimulating Hormone (TSH) in the carcinogenesis of differentiated thyroid carcinoma has already been demonstrated [47], but little is known about its relationship with tumor enlargement during AS. Currently, no controlled and randomized studies are available to determine the benefits of TSH suppression therapy in active surveillance. On one hand, Sugitani et al. [48] found no association between TSH levels and an increase in tumor diameter during AS of 323 patients with PMC after a mean follow-up of 6.5 years. In contrast, Kim et al*.* [31] found that one-third of patients who developed an increase in tumor volume of more than 50% during AS were classified into the upper tercile of normal TSH level ​​(2.3–4.5 mUI/mL). Moreover, this study estimated that serum TSH levels ​​greater than 2.5 mUI/L could be considered a progression-predictive cutoff for the target TSH level during AS [31]. Recently, the same group reported that age might have a relationship with TSH levels considering tumor growth. In this investigation, the authors found that TSH levels in the superior tercile (2.4 to 4.4) were associated with an impact on the growth of more than 30 times in those patients younger than 40 y.o. [49]. In agreement with these findings, Ito et al. performed a multivariate analysis of 92 patients from 2705 under active surveillance who showed an increase in tumor diameter. The group found that younger than 40 years, tumor size ≥ 9 mm, and TSH values ≥ 3 above the lower limit of normal were significant variables related to tumor enlargement [50].

According to Japanese practices, keeping the TSH level at a low-normal level might be beneficial in young patients because of their higher risk of tumor enlargement [37]. Since the results remain conflicting, presumably due to the progression parameters used in each study, levothyroxine therapy and the target TSH levels in patients under AS will be at the clinician’s discretion.

#### Pregnancy

Pregnancy is a known predisposing condition to increased thyroid gland stimulation through exponential secretion of human chorionic gonadotropin, among other hormonal factors [51]. The risk of potential tumor progression is not well established in prospective studies on active surveillance. In a cases series report, from fifty-one labors, four patients (8%) showed enlargement of PMC by ≥ 3 mm; one patient (2%) showed a decrease, and the remaining forty-four patients (45 events, 90%) showed stable disease. None of the patients had a novel appearance of lymph node metastases during pregnancy. After delivery, the PMC of one of these four patients that experienced progression remained stable, and another showed a decrease in PMC size [51]. Another study showed, from 9 pregnant women with PMC under AS, tumor enlargement was detected in 4 (44.4%), whereas it was observed only in 3 (11.1%) of the 27 patients in the control group [52].

Although the frequency of tumor growth during nine months of pregnancy was similar to that reported in non-pregnant patients over ten years, pregnant women or those who are planning pregnancy could be considered appropriate for AS since rescue surgery after childbirth can solve an eventual progression [43].

#### Molecular profile

At present, there are no studies that determined reliable molecular markers that predict progression in low-risk thyroid tumors. *BRAF V600E* mutations are known to be present in more than 50% of PMCs [9, 53], so their isolated presence does not constitute a contraindication for active surveillance [53]. Some authors recommend excluding from AS those tumors with a combination of driver mutations, e.g. *BRAF V600E* + *TERT or RAS* + *TERT* since they were associated with a worse prognosis in patients with PTC [16, 40, 54]. Nonetheless, Yabuta et al. [55] compared the incidence of BRAF mutations in surgical specimens of PMC presenting with lymph node metastases and increased tumor diameter with those who did not show progression, being similar among the three groups (80%, 70%, and 64%, respectively). In addition, he found no mutations in the *TERT *promoter. According to the available data, the cost-effectiveness of molecular markers in low-risk thyroid tumors remains controversial and will probably be an interesting topic of future research.

### Tumoral growth kinetics

As previously mentioned, most historical publications of AS have described 3 common tumor volume kinetic patterns: (i) a steady, exponential increase in tumor volume, (ii) a quiescent phase of apparently stable tumor volumes, and (iii) a decrease over time [19]. However, Ito et al. were the first author to show differential patterns of growth: a group of patients in whom tumor volume doubling rates declined after the first exponential growth (≥ 3 mm in diameter or ≥ 50% in volume). This may suggest that a continued exponential increase in tumor volume may not be inevitable [56]

**Fig. 2:**
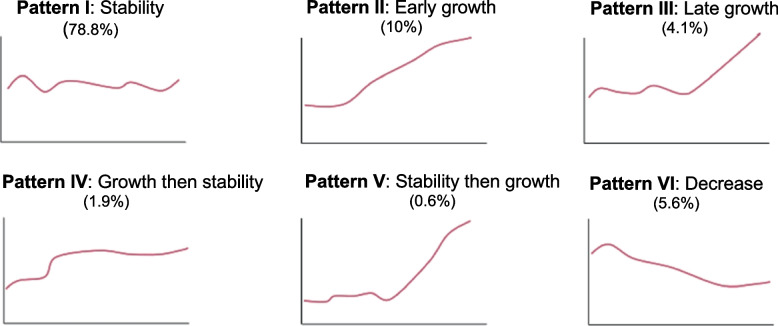
Different patterns of growth occurred in 483 patients under AS [57]

More recently, Tuttle et al*.* described these 6 different patterns of growth occurring in 483 patients under AS. At 5 years, most tumors demonstrated stability (78.8%, Pattern I) with 10.0% showing early growth (Pattern II), 4.1% late growth (Pattern III), 1.9% growth then stability (Pattern IV), 0.6% stability then growth (Pattern V) and 5.6% with a decrease in tumor volume (Pattern VI). Tumor volume doubling time during exponential growth significantly differed across the kinetic patterns, with median values of 2.4 years, 7.1 years, and 3.3 years for Patterns II, III, and IV, respectively (*p* < 0.01) [57] (Fig. 2).

These authors conclude that the tumor volume kinetic patterns should be added to the classical clinical framework that usually considers tumor size, tumor location, and patient preference.

### Adverse events and medical costs of thyroid surgery

When active surveillance is usually offered to the appropriate candidate, it is essential to clearly inform not only about the outcomes reported by different experiences worldwide but also the adverse events related to thyroid surgery to ensure the patient has complete information for adequate decision-making. The adverse events after total or subtotal thyroidectomy may be transient or permanent with a negative impact on the quality of life [29]. In a multicenter study that included 14,934 patients undergoing thyroidectomy, the overall complication rate was 17.4%, 7% of which were permanent [58]. Furthermore, we recently analyzed the adverse events of 135 patients who were candidates for active surveillance but decided to undergo immediate surgery. We found that a quarter of the patients had postoperative adverse events and about 10% of these were permanent [25]. In addition, our study also illustrated that the frequency of postoperative adverse events tends to decrease significantly if surgery is performed by high-volume surgeons [25, 59, 60]. On the other hand, lobectomy has a risk of permanent hypocalcemia and recurrent laryngeal nerve injury remarkably lower [25, 59]. Although it should be the surgery of choice for low-risk intrathyroidal tumors smaller than 4 cm in diameter [9], in some settings it is infrequently applied [25]. In the above-mentioned Argentinian experience, most patients were referred to lobectomy for a low-risk PTC (that is, the remaining 75% who did not accept AS) and finally underwent total thyroidectomy. This approach was decided according to the preference of the patient and the surgeon or to new opinions of other endocrinologists, who continue to choose this approach as the preferred option when they are faced with the diagnosis of any malignant thyroid tumor [25]. Finally, after a lobectomy, some studies informed that one out of five patients required hormonal replacement with levothyroxine [61], and a considerable proportion of patients referred to have neurocognitive and mood impairments [62].

The frequency of postoperative thyroid surgery adverse events is summarized in Table 2.

**Table 2:** Postoperative adverse events of thyroid surgery [25, 58]

Postoperative adverse event Frequency (%)	All risk PTC patients n: 14,934		Low-risk PTC patients n: 135	
	**Total Thyroidectomy**	**Lobectomy**	**Total Thyroidectomy**	**Lobectomy**
Total adverse events	N/A		*n* = 33 (24.4%)	
Permanent adverse events			*n* = 13 (9.6%)	
Hypoparathyroidism	10%		17%	
Transient	14%	0,4%	9.7%	0%
Permanent	2,2%	0,07%	5.2%	
Partial^a^			2.2%	
Parathyroid in surgical piece (without Hipoparathyroidism)			3.8%	
Vocal cord paralysis	3.4%		3%	
Transient	2,4%	1,4%	0.7%	0%
Permanent	1,3%	0,6%	2.2%	
Bilateral	0,6%	0%	0.7%	
Hypothyroidism	N/A		100%	50%
Hematoma	1,6%	0,4%	1,5%	0%
Surgical wound infection	0,4%	0,13%	0,7%	0%
Keloid scar	N/A		2,2%	0%
External superior laryngeal nerve injury	0,4%		N/A	
Thoracic duct injury	0,2%			
Ulnar nerve injury	0,2%			
Other^b^	0,9%			

^a^ Defined as a clinical disorder with normal serum parathormone but a requirement for ongoing calcium and calcitriol supplementation in order to avoid hypocalcemia and its symptoms for more than 6 months

^b^ Other complications: Claude-Bernard-Horner syndrome, punctate keratitis, alterations caused by neck hyperextension during surgery: vertigo, headache, nausea

When different management alternatives are offered to patients with low-risk PTC, medical costs should also be considered in order to balance the best decision. While global socioeconomic disparities demonstrate diverse healthcare system financial challenges, several investigators worldwide agreed on the high medical costs associated with the diagnosis and treatment of thyroid cancer. In this regard, it is projected that in the United States, costs will increase to USD 3.55 billion by 2030 [63]. Meanwhile, in Asia, some authors have compared the total cost of two groups of patients with thyroid microcarcinomas who underwent immediate surgery versus AS for ten years [64, 65], and they found that the total cost in ten years of immediate surgery was 4 to 6.5 times higher than AS. Furthermore, including the estimated price of an eventual deferred surgery during AS, the total cost of the immediate surgery was still 4.1 times more expensive than AS during the same period of time [64, 65]. A similar analysis was performed in Latin America, finding that lobectomy and total thyroidectomy resulted in 3 and 4 times higher, respectively than implementing a ten-year prospective active surveillance [39]. Finally, recent studies attempted to estimate the cost-effectiveness of both practices and their long-term financial outcomes. Thus, an American analysis revealed that, in this specific context, lobectomy was cost-effective in middle-aged patients (40–69 years) and active surveillance in patients aged 69 years or older [66]. On the other hand, the South Korean MAeSTro prospective study estimated that the initial cost of AS is 5.6 times lower than that of lobectomy, while the 10-year cumulative costs of AS and lobectomy would be similar [67]. While both studies reported interesting data, it is probably not accurately applied in different settings, especially in the long term, since costs may be affected by national health insurance coverage, the interval of thyroid ultrasound during follow-up, and the differences to access to health care in each country.

### Acceptance of AS and real-life application

Although there have been three decades of cumulated experience in active surveillance in low-risk PTC, acceptance toward performing AS is usually difficult for some patients in clinical practice. The adherence to AS may be difficult to be predicted since it is strongly influenced by the values and preferences of the patients, and some of them may desist from AS due to the anxiety of the burden of living with cancer [16]. In a study conducted in the United States, out of 10,795 eligible patients, only 15.5% accepted AS [68]. Meanwhile, in Argentina, only 34 (25%) of 136 patients candidates for active surveillance accepted this approach, and around 10% of those abandoned it due to anxiety [25]. Nevertheless, the trend of acceptance is on the rise in some settings [69, 70]. For example, in the early days of AS in Japan, the proportion of patients who chose this alternative was only 30% and today is almost tripled [69]. The authors believe these differences are accompanied by the increasing acceptance of the approach by the medical community [69], but probably, a selection bias plays a role that makes this variability remains uncertain. It is within this context that, despite the proven insidious natural history of low-risk papillary carcinomas and the benefits of their observation in terms of the avoidance of unnecessary surgeries, their adverse events, and the high costs involved, the application of active surveillance in a real-life scenario is subject not only to the availability of a specialized medical team but also its acceptability, elicited by minimalism and compliance.

The frequency of active surveillance acceptance in different series is summarized in Table 3.

**Table 3:** Reports about the frequency of active surveillance adherence as an elective approach in low-risk PTC among countries

Country	n	Year	AS acceptance (%)	Withdrawal (%)
**Japan** [69]	4023	1993–1997	30	N/A
		2014–2016	88	
**USA** [68]	10,795	2017	15.5	N/A
**S. Korea** [70]	439	2018	34	19
	751	2022	64.1	N/A
**Argentina** [22]	164	2018–2020	25	10
**Brazil** [24]	77	2018	84	N/A
**Canada** [71]	100	2020	70	N/A

### New insights in AS: is it possible in indeterminate nodules?

In comparison with a Bethesda category V/VI thyroid nodule, FN/SFN (follicular neoplasm or suspicious for a follicular neoplasm) presents a markedly lower risk of malignancy, but in case it is, the histological diagnosis may result in a follicular carcinoma (including a Hürthle cell carcinoma), with a potentially more aggressive clinical behavior [72, 73]. Molecular tests may be used today for the risk assessment of these nodules to avoid unnecessary surgery, but often they may not be conclusive and not widely available [74]. Thus, a recent study prospectively evaluated 155 patients with Bethesda category IV thyroid nodules in which immediate molecular testing and/or thyroid surgery were offered [75]. Fifteen percent of them underwent active surveillance due to the failure to afford molecular testing, rejected the surgery, had a high surgical risk, or had other disorders/comorbidities which needed to be addressed with higher priority. Among these patients, the frequency of tumor enlargement was 14% (*n* = 3), after a median of 42 months (range, 7–72) of follow-up, without any evidence of lymph node or clinical distant metastases development. Deferred surgery was performed in 4 patients (17%) after a median of 24 months (range, 12–48) of AS. Follicular adenoma was diagnosed in three and a follicular variant of papillary thyroid carcinoma in one patient, all of them without evidence of disease after 12 months of follow-up [75]. Similarly, another study showed tumor growth in 2 of 15 patients (14.2%) with unoperated Bethesda IV thyroid nodules. Thyroidectomy was performed in 5/15 patients (33%) after a mean follow-up of 5 years but conversely, cancer was diagnosed in 3/5 (60%) [76]. Since these findings showed that most of these patients had excellent outcomes, AS could probably be a valid alternative in these low-risk tumors, mainly in settings with a low prevalence of thyroid cancer, the access to lobectomy and molecular testing is limited.

## Conclusions

The high prevalence of low-risk papillary thyroid, its indolent course, and the excellent outcomes of active surveillance led to this approach as a safe and feasible alternative. The high rate of adverse events and medical costs associated with surgery highlight the importance of considering this practice. The knowledge about the evolution of this group of patients for its proper selection leads towards active surveillance as a new paradigm approach of management in low-risk papillary thyroid carcinomas.

## Data Availability

Data sharing is not applicable to this article as no datasets were generated or analyzed during the current study.

## Abbreviations

AS: Active surveillance
FNAB: Fine-needle aspiration biopsy
FN/SFN: Follicular neoplasm or suspicious for a follicular neoplasm
TSH: Thyroid stimulating hormone
PMC: Papillary microcarcinoma
PTC: Papillary thyroid carcinoma
